# Effective induction therapy for anti-SRP associated myositis in childhood: A small case series and review of the literature

**DOI:** 10.1186/s12969-017-0205-x

**Published:** 2017-10-31

**Authors:** E. L. Binns, E. Moraitis, S. Maillard, S. Tansley, N. McHugh, T. S. Jacques, L. R. Wedderburn, C. Pilkington, S. A. Yasin, K. Nistala, on behalf of the UK Juvenile Dermatomyositis Research Group (UK and Ireland), Kate Armon, Joe Ellis-Gage, Holly Roper, Vanja Briggs, Joanna Watts, Liza McCann, Ian Roberts, Eileen Baildam, Louise Hanna, Olivia Lloyd, Susan Wadeson, Phil Riley, Ann McGovern, Clive Ryder, Janis Scott, Beverley Thomas, Eslam Al-Abadi, Sue Wyatt, Gillian Jackson, Tania Amin, Mark Wood, Vanessa Van Rooyen, Deborah Burton, Joyce Davidson, Janet Gardner-Medwin, Neil Martin, Sue Ferguson, Liz Waxman, Michael Browne, Mark Friswell, Helen Foster, Alison Swift, Sharmila Jandial, Vicky Stevenson, Debbie Wade, Ethan Sen, Eve Smith, Lisa Qiao, Stuart Watson, Claire Duong, Helen Venning, Rangaraj Satyapal, Elizabeth Stretton, Mary Jordan, Ellen Mosley, Anna Frost, Lindsay Crate, Kishore Warrier, Stefanie Stafford, Lucy Wedderburn, Clarissa Pilkington, Nathan Hasson, Sue Maillard, Elizabeth Halkon, Virginia Brown, Audrey Juggins, Sally Smith, Sian Lunt, Elli Enayat, Hemlata Varsani, Laura Kassoumeri, Laura Beard, Katie Arnold, Yvonne Glackin, Stephanie Simou, Beverley Almeida, Kiran Nistala, Raquel Marques, Claire Deakin, Stefanie Dowle, Charis Papadopoulou, Cerise Johnson-Moore, Emily Robinson, Kevin Murray, John Ioannou, Linda Suffield, Muthana Al-Obaidi, Helen Lee, Sam Leach, Helen Smith, Anne-Marie McMahon, Heather Chisem, Ruth Kingshott, Nick Wilkinson, Emma Inness, Eunice Kendall, David Mayers, Ruth Etherton, Danielle Miller, Kathryn Bailey, Jacqui Clinch, Natalie Fineman, Helen Pluess-Hall, Lindsay Vallance, Louise Akeroyd, Alice Leahy, Amy Collier, Rebecca Cutts, Emma Macleod, Hans De Graaf, Brian Davidson, Sarah Hartfree, Danny Pratt

**Affiliations:** 1 Infection, Immunity, Inflammation and Programme, Great Ormond Street, UCL Great Ormond Street, Institute of Child Health, London, UK; 2 Department of Pharmacy and Pharmacology, University of Bath, Bath, UK; 3 Developmental Biology and Cancer Programme, UCL Institute of Child Health, London, UK; 4 Rheumatology Unit, Great Ormond Street Hospital for Children, London, UK; 5 UCL Division of Medicine, London, UK; 6 NIHR Biomedical Research Centre at Great Ormond Street Hospital for Children NHS Foundation Trust, University College London, London, UK; 7 Arthritis Research UK Centre for Adolescent Rheumatology at UCL, UCLH and GOSH, London, UK; 8 Department of Histopathology at Great Ormond Street Hospital for Children NHS Foundation Trust, University College London, London, UK

## Abstract

**Background:**

Anti-Signal Recognition Particle associated myopathy is a clinically and histopathologically distinct subgroup of Juvenile Idiopathic Inflammatory Myositis, which is under-recognised in children and fails to respond to conventional first line therapies. We present three cases where remission was successfully induced using combination therapy with intensive rehabilitation.

**Case presentations:**

Three new patients are reported. All 3 cases presented with profound, rapid-onset, proximal myopathy and markedly raised CK, but no rash. Histology revealed a destructive myopathy characterized by scattered atrophic and necrotic fibres with little or no inflammatory infiltrate. All 3 patients responded to induction with cyclophosphamide, IVIG and rituximab, in conjunction with intensive physiotherapy and methotrexate as the maintenance agent. Our patients regained near-normal strength (MMT > 70/80), in contrast with the current literature where >50% of cases reported severe residual weakness.

A literature search on paediatric anti-SRP myositis was performed to June 2016; PubMed was screened using a combination of the following terms: signal recognition particle, autoantibodies, antibodies, myositis, muscular diseases, skeletal muscle, childhood, paediatric, juvenile. Articles in a foreign language were excluded. Nine case studies were found.

**Conclusion:**

This paper supports the hypothesis that anti-SRP myositis is distinct from other JIIM. It is an important differential to JDM and should be considered where there is severe weakness without rash or if highly elevated muscle enzymes (CK > 10,000 U/l) are found. Early identification is essential to initiate aggressive medical and physical therapy. Greater international collaboration and long-term follow-up data is needed to establish the most effective treatment strategy for this rare group of patients.

## Background

Juvenile Idiopathic Inflammatory myopathies (JIIM) are a heterogeneous group of autoimmune diseases that were originally categorized by their clinical phenotypes. In recent years, it has become clear that there is scope for further sub classification using serological phenotypes defined by Myositis-Specific Antibodies (MSA’s) and Myositis-Associated Antibodies (MAA’s) [1–8]. Anti-Signal Recognition Particle (anti-SRP) is an MSA which has been well described in the adult population but rarely in children. JIIM associated with anti-SRP antibodies has been characterized by severe, progressive proximal muscle weakness, minimal skin involvement, and markedly raised CK (usually more than 40 times greater than the upper limit of normal) with high levels of disability [2].

Thus far, the paediatric literature has demonstrated a picture consistent with the adult population, however only a handful of cases have been reported [9–15]. Currently the limited number of cases precludes definitive conclusions to guide clinicians in their management and thus a variety of therapies have been trialled on an individual basis, with variable success.

In this paper, we discuss three new cases of anti-SRP myositis and relate this to the published literature. Our report highlights the use of an aggressive induction regime where standard therapy failed, including combination of intravenous immunoglobulin (IVIG), cyclophosphamide and rituximab, with intensive physical therapy, which resulted in positive outcomes in each case.

## Case presentations

Three new cases are reported. All patients were included following full, informed parental consent and age appropriate assent. The study was approved by the North Yorkshire Multi Centre Research Ethics Committee, Ref: MREC/1/3/22.

A literature search on paediatric anti-SRP myositis was performed to June 2016; PubMed was screened using a combination of the following terms: signal recognition particle, autoantibodies, antibodies, myositis, muscular diseases, skeletal muscle, childhood, paediatric and juvenile. Articles in a foreign language were excluded. Nine case studies were found.

### Case reports

#### Case study 1

A previously healthy, 14-year-old female of Afro-Caribbean descent, born in the UK, presented with a 4 month history of progressive severe proximal muscle weakness, myalgia, ankle swelling and headaches. A fortnight prior to these symptoms she recalled a coryzal illness.

Examination revealed a profound, weakness; with a MMT8 score of 34/80 and a CMAS score 21/53, in a proximal distribution. There were no skin signs.

**Fig. 1:**
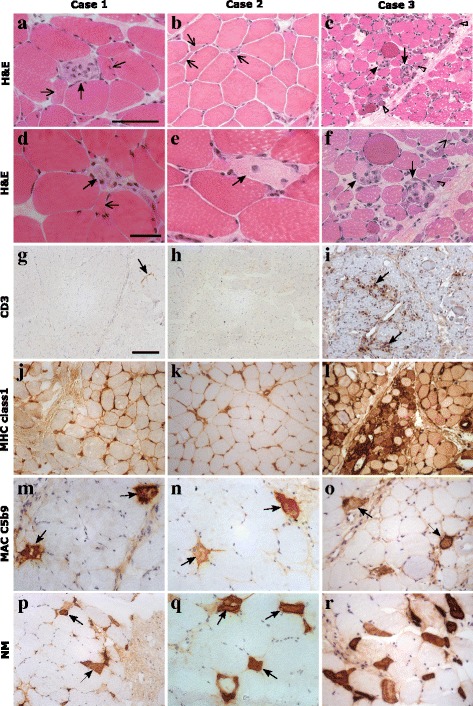
Muscle histology of all 3 SRP cases: **a**-**f** haematoxylin and eosin (H&E) stain showing scattered necrotic fibres (arrows) and atrophic fibres (open arrows) across the biopsies. **c** and **f** Numerous regenerating fibres (open arrow heads) could also be seen in case 3. **g**-**i** there was very little staining for CD3 in case 1 (arrow) and none in case 2 **h**. Moderate levels of CD3 were seen in case 3 in the endomysial compartment (**i**, arrows). **j**-**l** there was diffuse MHC class 1 up regulation in all 3 cases, but case 3 had higher levels at the sarcolemma and within fibres. **m**-**o** MAC C5b9 stained necrotic fibres in all cases (arrows) and also some endomysial capillaries (not shown). **p**-**r** there was scattered expression of neonatal myosin in the muscle fibres of case 1 and 2 (**p** & **q**, arrows) and expression in numerous fibres in case 3 **r**. Scale bars: a, b, f, m-o, q and r – 100 μm. c, g-l, p - 250 μm. d and e - 50 μm

Investigations showed a markedly raised CK of 23,111 U/l (50–240 U/l), LDH of 4553 U/l (380–640 U/L) and ALT of 300 U/I (10–45 U/l). EMG was compatible with polymyositis and MRI leg revealed widespread symmetrical signal abnormality. Thigh muscle biopsy showed destructive necrotising myopathy with minimal inflammatory infiltrate (Fig. 1). Chest X-ray was normal but PFT’s revealed a reduced DLCO/VA of 69%. CT chest, ECG and echocardiogram were normal. Video fluoroscopy demonstrated moderate oro-pharyngeal dysphagia with reduced bulbar musculature.

**Fig. 2:**
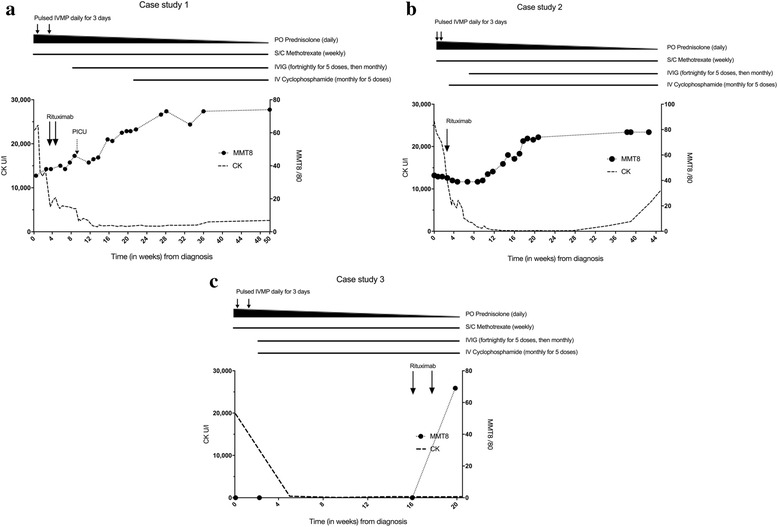
Summary of treatment and clinical course for Case 1 (**a**), Case 2 (**b**) and Case 3 (**c**). Line chart of muscle strength (dotted line with circles, Manual muscle testing, MMT, range 0–80, low numbers indicate weakness) and creatine kinase (CK, intermittent line) from diagnosis. Bold arrows indicate treatment with Rituximab. Duration of treatment is represented by horizontal lines above chart. Short vertical lines indicate treatment with high dose intravenous methylprednisolone (IVMP). Narrowing of the top horizontal line represents tailing of the oral corticosteroid dose (PO prednisolone)

Initial medical treatment was with high dose pulsed IVMP, 1 g daily for 3 days, followed by daily 1 mg/kg prednisolone and weekly subcutaneous methotrexate at 15 mg/m2. An improvement was noted in CK levels but she remained profoundly weak. In week 4 of admission she received a further course of IVMP and had her first dose of rituximab at 750 mg/m2, which was repeated 2 weeks later. Bloods at week 8 confirmed B cell depletion. Towards the end of week 8 the patient developed fever and respiratory distress and was admitted to the PICU for 5 days for treatment of CMV pneumonitis and CPAP support. In week 9, whilst in PICU, she commenced IVIG 2 g/kg for treatment of her pneumonitis as well as her myositis. She continued IVIG 2 g/kg fortnightly for 5 doses then monthly dosing. Following recovery from her respiratory infection Patient 1 had a steady improvement in her muscle strength which plateau’d at an MMT8 score of 60. MRI scans of her thighs at week 19 continued to show active disease and muscle atrophy therefore in week 20 she was commenced on pulsed intravenous cyclophosphamide at 500 mg/m2, monthly, for 5 doses. She was discharged at 22 weeks on methotrexate 25 mg weekly and prednisolone 5 mg daily with a plan to complete further doses of IVIG and cyclophosphamide as an outpatient. Her MMT8 score at discharge was 61/80, which increased to 74/80 at 12 months from presentation (Fig. 2a). Her CMAS score at 12 months was 48/53, by which time she was at school full-time and able to re-join her physical education and dance classes. CK at discharge was 2582 U/l. Her discharge maintenance treatment was monthly IVIG 2 g/kg, weekly methotrexate 15 mg/kg and 10 mg prednisolone with a plan to wean as per protocol.

#### Case study 2

A 13-year-old female of Afro-Caribbean descent, presented with a 4-week history of myalgia, proximal muscle weakness, limb and peri-orbital swelling and shortness of breath on exertion. Her symptoms were preceded by a mild coryzal illness and urticarial rash.

Examination revealed profound, asymmetrical proximal weakness preventing ambulation with an MMT8 score of 44/80 and CMAS of 31/53. She had Raynaud’s phenomena but no cutaneous features of JDM. Cardiovascular, respiratory and SLT exam were normal.

CK was elevated at 25,937 U/l (50-240 U/l) with an AST of 606 IU/l (10–40 U/l) and LDH of 2128 IU/l (380–640 U/l). Urine was positive for myoglobin but renal function was preserved. MRI thigh revealed evidence of myositis and fasciitis and EMG was consistent with a myopathic process. Muscle biopsy showed a necrotising myositis (Fig. 1). PFT’s revealed a DLCO/VA ratio 43%. Echo and ECG were normal.

Initial medical treatment was 2 courses of IVMP 1 g daily for 3 days 10 days apart, followed by oral prednisolone, initially at 1 mg/kg, and weekly sub-cutaneous methotrexate at 15 mg/m2. During this time her weakness progressed with a trough MMT8 of 39/53. In her 3rd week of admission she had a 750 mg/m2 dose of rituximab, together with 500 mg/m2 cyclophosphamide. She developed a groin abscess and as she had already depleted her B cells, no further doses of rituximab were given and cyclophosphamide was temporarily placed on hold. In week 6, she was commenced on monthly IVIG 2 g/kg and at week 10, she restarted cyclophosphamide for a total of 5 doses. At 17 weeks, she had improved significantly and was discharged on weekly methotrexate 15 mg/m2, monthly IVIG 2 g/kg and daily prednisolone 5 mg, to wean as per protocol. Her CK on discharge was 110 U/l, CMAS score was 31/53. At her 4-month follow-up, she had returned to school with an MMT8 score of 78/80 (Fig. 2b).

#### Case study 3

The third case is an 11-year-old female of West African descent. She presented with profound proximal myopathy, myalgia and arthralgia following an upper respiratory tract infection a week previously. Examination revealed an extreme proximal weakness scoring 0/53 CMAS and an un-recordable MMT8. There was no joint restriction or inflammation and no rash. She described no difficulty in swallowing but her voice had developed a nasal quality.

CK was elevated at 19,808 U/l (50–240 U/l), AST of 512 IU/l (10–40 U/l), and LDH of 3567 IU/l (380–640 U/l). MRI of the thighs showed diffuse patchy oedema in all muscle groups and EMG was consistent with myositis. Muscle biopsy revealed a destructive myopathy (Fig. 1). CXR was normal but CT thorax showed ground glass opacification and video fluoroscopy was consistent with chronic micro-aspiration therefore she received nasogastric feeding and soft diet as an inpatient. Echo was normal. PFTs showed FEV1 59%, FVC 58% predicted, whilst repeat CT chest at 4 months was normal. A respiratory consult suggested that abnormal PFTs represented weakness rather than interstitial lung disease.

Medical treatment began with 2 courses of pulsed IVMP 1 g over 3 days, followed by daily oral prednisolone, initially at 2 mg/kg, and methotrexate 15 mg/m2. At week 2 she had her first dose of cyclophosphamide 500 mg/m2, monthly dosing for 5 doses, together with IVIG at 1 g/kg initially at fortnightly dosing. Her cyclophosphamide dose was decreased to 375 mg/m2 after 2 doses due to microscopic haematuria. Muscle strength began to improve but regressed again after a period of reduced access to physiotherapy due to chickenpox contact. Her medical treatment was subsequently escalated and, at week 16, she received her first dose of rituximab, 750 mg/m2, with a further dose a fortnight later. Following this she had a steady improvement and was discharged after a 19-week admission on prednisolone 15 mg daily and methotrexate 20 mg weekly, with an MMT8 of 69/80 and CMAS of 49/53 (Fig. 2c). Her CK at discharge was 269 U/L. Discharge maintenance therapy was weekly methotrexate 15 mg/kg, monthly IVIG 2 g/kg and daily 10 mg prednisolone, with a plan to reduce steroids as per local protocol.

#### Approach to physical therapy

All 3 girls in our series commenced intensive physiotherapy from the day of admission. Initially, as they were unable to move their limbs with anti-gravity movements, therapy was centred round stretching muscle length to regain full joint range of movement, taking to care to avoid overstretching and hypermobility. Care had to be taken when our patients had improved enough to walk, as extreme core muscle and gluteal weakness posed a significant risk of falls and injury. Reduced head control was an issue and the girls were provided with reclining wheelchairs with headrests so they could engage in activities without muscle fatigue.

Initially the physiotherapy programme consisted of active-assisted exercises targeting individual muscles and avoiding complex movements that require multiple muscles working effectively. Recovery of muscle strength was extremely slow with the proximal muscle groups; neck flexors, abdominals, gluteals and vastus medialis took 2–3 months before recovery of functional strength began.

### Literature review

The PubMed database was studied up to May 2016. Nine cases of Anti-SRP myositis in children, aged 16 and under, were identified [9–15]. Eight cases were female and 1 male. The mean age was between 11 and 12 with the youngest patient aged 6 [9]. Ethnicity was known for 8/9 patients [9–14]. The majority of cases were non-Caucasian; 4 were Japanese [9–12] 3 were African- American [13], one was Alaskan [14]. Chronobiological data was collected in half the cases; these occurred between October- February [13, 14]. Half reported a preceding infection in the months prior to onset [11–13].

Severe proximal weakness in the absence of typical skin findings of JDM was a universal feature. Common associated symptoms were Raynaud’s (3/8) [13, 15], arthritis (3/8) [13, 14] and dysphagia (3/8) [11–13, 15]. 3 children had signs of restrictive lung disease on PFT’s [13–15], although as seen in our cases this may reflect respiratory muscle weakness rather than ILD which is rare in adult anti-SRP syndrome. Cardiac involvement was noted in 2 patients with LVH on Echo [13]. All patients had a muscle biopsy showing necrosis and in 3 cases this was with a lymphocytic infiltrate [13, 15].

Treatment schedules varied widely: All patients were initially treated with steroids and 5/9 with methotrexate ^[913–15]^ with a universally poor response. Three patients showed improvement with addition of IVIG [13–15], however 2 of these subsequently relapsed. Rituximab was used as an induction agent in one case with a good response [15].

Five cases were treated with cyclophosphamide: 1 case developed ovarian failure and treatment was therefore discontinued [13] and 3 cases were poor responders [9, 13]. One patient had a dramatic improvement in muscle strength with combination induction therapy using plasma exchange and methylprednisolone followed by IV cyclophosphamide and azathioprine [11, 12]. Azathioprine has also been used successfully as a maintenance agent in combination with IVIG and levflunomide following induction with rituximab [15]. A poor response was documented in the 2 cases where Azathioprine was used to induce remission [13, 15]. Infliximab was found to be ineffective in 3 patients [13]. Cyclosporine had a modest effect in one case and no effect in another [13]. Tacrolimus was used in 2 patients; it was discontinued in one due to haemolytic uraemic syndrome [13] and was ineffective in the other [9]. Mycophenalte Mofitil (MMF) was used in one patient with a modest effect [13].

Long-term outcomes in the existing literature are variable but generally poor. 3/9 patients achieved remission and remained clinically well at the time of publication [11, 12, 14, 15]. One of these had a period of relapse but subsequently went back into remission [15]. Two further cases showed initial improvement but relapsed following an infection [9, 14, 15]. Over 50% of cases had severe residual weakness [9, 10, 13] with 80% of that group remaining wheelchair bound [9, 10, 13].

## Discussion

Our report highlights three paediatric cases of anti-SRP necrotising myositis presenting at a single centre during 2014–15. All 3 cases were initially identified with Immunoblot and subsequently confirmed by immunoprecipitation. All cases were negative for other myositis specific and myositis associated antibodies. Given the rarity of published paediatric cases, our report helps confirm the clinical and histological findings in the paediatric age group and importantly, identifies an induction regime that was effective in all 3 patients.

The original Bohan and Peter [16] criteria for IIM have not been validated in the paediatric population and fail to specifically identify children with necrotising myopathies. From a clinical perspective, this distinction is essential as patients with anti-SRP myositis fail to respond to first line therapy for JDM and are at risk of poor outcomes.

Muscle biopsy histology alone is unlikely to accurately distinguish necrotising myopathies from JDM, as some cases of anti-synthetase associated myopathies can have necrotic features, and conversely anti-SRP biopsies may have an inflammatory infiltrate (Fig. 1, case 3). Interestingly, one of our cases had tubuloreticular inclusion bodies on electron microscopy – previously considered a characteristic feature of DM/JDM. All our patients were tested for anti-HMGCR by ELISA with no positive results [17]. To ensure the correct diagnosis of anti-SRP myositis, we would recommend that all patients diagnosed with Juvenile IIM be tested for MSAs including anti-SRP.

In all 3 of our patients, standard treatment with methotrexate and steroids failed to significantly improve muscle strength. Each patient subsequently responded to combination therapy with rituximab, cyclophosphamide and IVIG, together with a longer duration of intensive daily physical therapy. Although 2 of the more severe cases took between 6 (case 1) and 12 (case 3) months to regain MMT > 70 all 3 were able to regain independent mobility. Recent data from the RIM study suggests that the median time to achieve a 20% improvement in MMT8 with rituximab is 20 weeks [18]. The nature of our report precludes definitive conclusions on the comparison of combination therapy vs individual agents, however, it is notable that in all 3 of our cases an improvement in MMT8 occurred between 12 and 16 weeks, which is earlier than data for rituximab alone. Given that anti-SRP myositis is usually more resistant to therapy than other forms of adult and juvenile myositis, we suggest that our patient’s results reflect the combination of rituximab together with cyclophosphamide and IVIG rather than from one agent alone.

Currently there is a lack of data regarding long term outcomes of anti-SRP myositis. A recent large cohort study of 37 adult patients with anti-SRP myositis found that only 50% of their cohort reached near-full or full strength after 4 years of treatment and that most continued to have persistently elevated CK levels [19]. This study also documented worse outcomes in their younger patients.

In spite of the good initial response to combination therapy, two of our patients (case 1 and 2) had rising CK levels 7–8 months after presentation, once the prednisolone dose had fallen below 10 mg and they remained on MTX and IVIG alone. In both patients CK levels responded to an escalation in corticosteroids and re-dosing with rituximab. Long term follow-up of paediatric patients is needed to monitor for evidence of ongoing active disease after treatment.

## Conclusion

This paper supports the evolving hypothesis that Anti-SRP is clinically and histopathologically distinct from other JIIM. It is an important differential to JDM and should be considered where there is severe weakness with minimal rash or in patients with highly elevated muscle enzymes (CK > 10,000iu/l).

The current literature suggests that patients with anti-SRP antibodies do not respond to conventional first line therapies and have generally poor outcomes. We believe early identification is essential so as not to delay aggressive medical and physical therapy. Currently there is no consensus on the best therapeutic protocol for these children but our cases demonstrate that aggressive treatment with cyclophosphamide, rituximab and IVIG, in addition to standard therapy with methotrexate and corticosteroids can induce remission. We advocate for intensive physiotherapy as an integral therapeutic measure.

Adult data has shown that anti-SRP myositis is a chronic condition with ongoing high CK levels even in successfully treated patients [19]. More data is required to understand if this is also true of children.

Greater international collaboration and long-term follow-up data is needed to establish the most effective treatment strategy for this rare group of patients.

## Abbreviations

ALT: Alanine aminotransferase
AST: Aspartate aminotransferase
CK: Creatine kinase
CMAS: Childhood myositis assessment scale
CT: Computed tomography
DLCO/VA: Diffusing capacity of lungs for carbon monoxide/alveolar volume
DM: Dermatomyositis
ECG: Electrocardiogram
ECHO: Echocardiogram
EMG: Electromyography
FEV1: Forced expiratory volume 1
FVC: Forced vital capacity
ILD: Interstitial lung disease
IVIG: Intravenous immunoglobulins
IVMP: Intravenous methylprednisolone
JDM: Juvenile dermatomyositis
JIIM: Juvenile idiopathic inflammatory myopathies
LDH: Lactate dehydrogenase
LVH: Left ventricular hypertrophy
MAA: Myositis-Associated antibodies
MMF: Mycophenolate mofetil
MMT8: Manual Muscle Testing 8
MRI: Magnetic resonance imaging
MSA: Myositis-Specific antibodies
MTX: Methotrexate
PFT: Pulmonary function test
SLT: Speech and language therapy
